# Phosphatase-regulated recruitment of the spindle- and kinetochore-associated (Ska) complex to kinetochores

**DOI:** 10.1242/bio.026930

**Published:** 2017-10-05

**Authors:** Sushama Sivakumar, Gary J. Gorbsky

**Affiliations:** Cell Cycle and Cancer Biology Research Program, Oklahoma Medical Research Foundation, Oklahoma City, OK 73104, USA

**Keywords:** Mitosis, Spindle checkpoint, Cell cycle, Microtubules, Anaphase-promoting complex/cyclosome

## Abstract

Kinetochores move chromosomes on dynamic spindle microtubules and regulate signaling of the spindle checkpoint. The spindle- and kinetochore-associated (Ska) complex, a hexamer composed of two copies of Ska1, Ska2 and Ska3, has been implicated in both roles. Phosphorylation of kinetochore components by the well-studied mitotic kinases Cdk1, Aurora B, Plk1, Mps1, and Bub1 regulate chromosome movement and checkpoint signaling. Roles for the opposing phosphatases are more poorly defined. Recently, we showed that the C terminus of Ska1 recruits protein phosphatase 1 (PP1) to kinetochores. Here we show that PP1 and protein phosphatase 2A (PP2A) both promote accumulation of Ska at kinetochores. Depletion of PP1 or PP2A by siRNA reduces Ska binding at kinetochores, impairs alignment of chromosomes to the spindle midplane, and causes metaphase delay or arrest, phenotypes that are also seen after depletion of Ska. Artificial tethering of PP1 to the outer kinetochore protein Nuf2 promotes Ska recruitment to kinetochores, and it reduces but does not fully rescue chromosome alignment and metaphase arrest defects seen after Ska depletion. We propose that Ska has multiple functions in promoting mitotic progression and that kinetochore-associated phosphatases function in a positive feedback cycle to reinforce Ska complex accumulation at kinetochores.

## Abstract (precis)

**Summary:** Feedback between protein phosphatases and the spindle- and kinetochore-associated (Ska) complex regulates chromosome movement and the metaphase-to-anaphase cell cycle transition. This article has an associated First Person interview with the first author of the paper as part of the supplementary information.

## INTRODUCTION

At mitotic entry, protein kinases phosphorylate numerous substrates to trigger important events such as chromosome condensation, nuclear envelope breakdown, mitotic spindle assembly, and proper kinetochore-microtubule interactions (Bollen et al., 2009; Wurzenberger and Gerlich, 2011). Throughout mitosis and particularly at mitotic exit, these phosphorylations are opposed by phosphatases. In mammalian cells, protein phosphatase 1 (PP1) and protein phosphatase 2A (PP2A), in association with specific regulatory and targeting subunits, are thought to dephosphorylate many of the substrates targeted by mitotic kinases (Bollen et al., 2009).

The three proteins of the spindle- and kinetochore-associated (Ska) complex, Ska1, Ska2, and Ska3, exist as an obligate hexamer containing two copies of each (Gaitanos et al., 2009; Welburn et al., 2010). Depletion of any of the Ska proteins causes degradation of its partners, impaired chromosome alignment, and metaphase delay or arrest (Daum et al., 2009; Gaitanos et al., 2009; Hanisch et al., 2006; Redli et al., 2016; Schmidt et al., 2012; Sivakumar et al., 2014; Theis et al., 2009; Welburn et al., 2009). Both Ska1 and Ska3 bind to microtubules and promote chromosome movement (Abad et al., 2014, 2016; Schmidt et al., 2012). Ska1 also recruits PP1 to kinetochores (Sivakumar et al., 2016), and the Ska complex promotes binding of the anaphase-promoting complex/cyclosome (APC/C) to mitotic chromosomes (Sivakumar et al., 2014). Phosphorylation of Ska by Aurora B kinase inhibits its binding to kinetochores, but paradoxically, may also promote Aurora B accumulation on kinetochores and increase its kinase activity *in vivo* and *in vitro* (Chan et al., 2012; Redli et al., 2016).

Two isoforms of PP1 (PP1γ and PP1α) are concentrated at kinetochores and bind Knl1 and Ska1 (Liu et al., 2010; Sivakumar et al., 2016; Trinkle-Mulcahy et al., 2003, 2006). Kinetochore-associated PP1 appears to play important roles in stabilizing kinetochore-microtubule attachments and opposing spindle checkpoint signaling (Liu et al., 2010; Pinsky et al., 2006; Sivakumar et al., 2016; Vanoosthuyse and Hardwick, 2009).

The PP2A holoenzyme is a hetero-trimer composed of a scaffolding A subunit, regulatory B subunit and catalytic C subunit (Janssens et al., 2008). The B subunits are classified into three sub-families termed B (PR55/B55), B′(PR61/B56) and B″(PR72) (Bollen et al., 2009; Janssens et al., 2008). Plk1 phosphorylation of BubR1 recruits PP2A-B56 to kinetochores in prometaphase (Foley et al., 2011; Suijkerbuijk et al., 2012). At metaphase, PP2A-B56 levels diminish at kinetochores while PP1 increases, suggesting that kinetochore-microtubule interactions are stabilized by PP2A-B56 in prometaphase and by PP1 at metaphase. In agreement with this idea, depletion of PP2A shows stronger impairment of chromosome alignment compared to depletion of PP1 (Foley et al., 2011; Liu et al., 2010).

In this study, we show that PP1 and PP2A phosphatases promote Ska recruitment to kinetochores. These results corroborate and extend previous work (Redli et al., 2016). Forced targeting of PP1 to kinetochores partially rescues defects caused by Ska3 depletion. We propose a feedback mechanism in which the Ska complex recruits PP1 to kinetochores at metaphase which further recruits Ska to stabilize kinetochore-microtubule attachments and initiate anaphase.

## RESULTS AND DISCUSSION

### Phosphatases promote accumulation of Ska at kinetochores

**Fig. 1.**
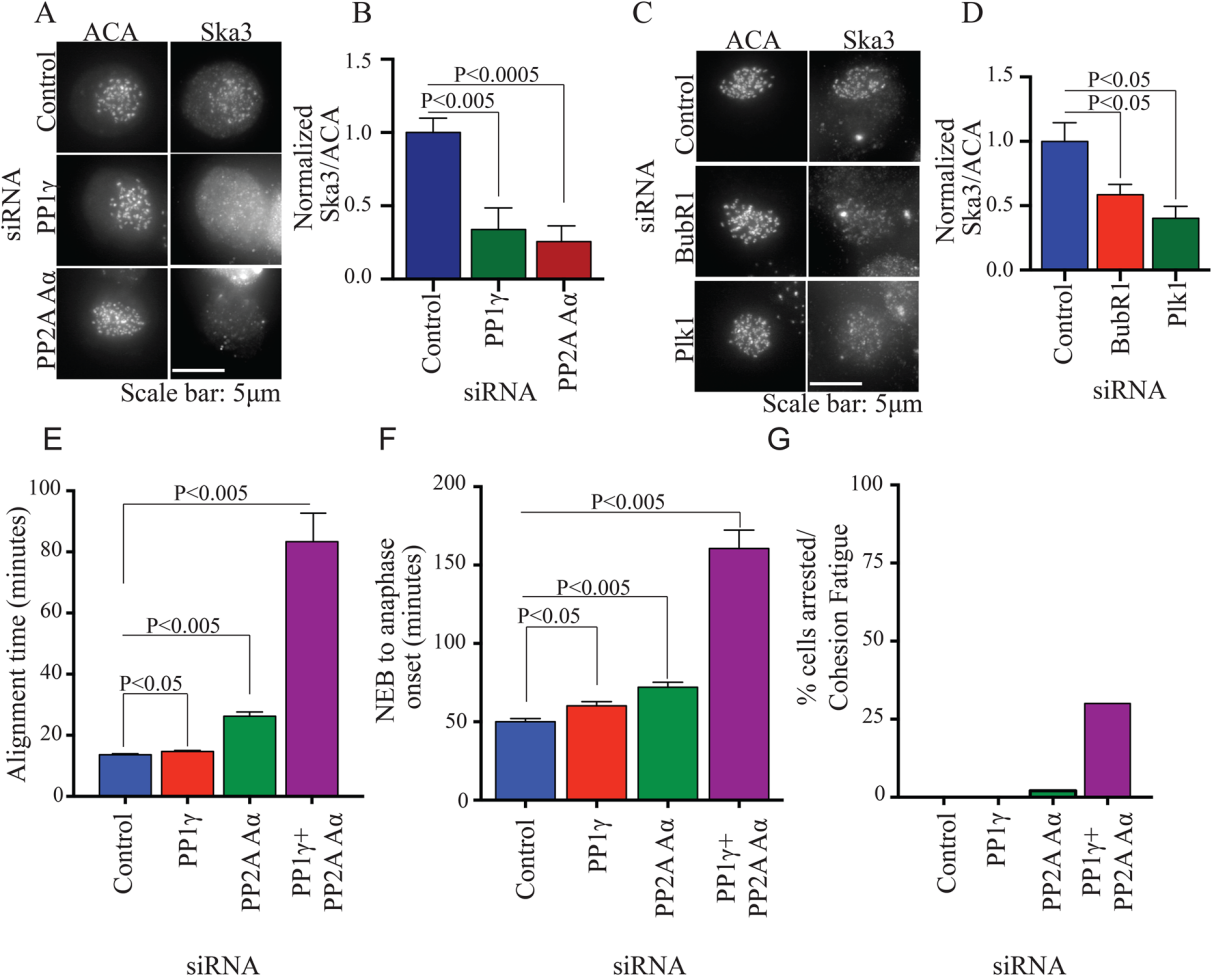
**Phosphatases PP1 and PP2A promote Ska recruitment and normal progression through mitosis.** (A) HeLa cells grown on coverslips were transfected with control, PP1γ or PP2A Aα siRNA. 45 h after transfection, cells were treated with 3.3 μM nocodazole for 3 h and then prepared for immunofluorescence. Ska3 at kinetochores was quantified. PP1γ or PP2A Aα depletion reduced Ska3 at kinetochore. (B) Bar graph depicting mean fluorescence intensity of Ska3 at kinetochores normalized to anti-centromere antibody (ACA). (C) HeLa cells grown on coverslips were transfected with control, Plk1 or BubR1 siRNA. 45 h after transfection cells were treated for 3 h with 3.3 μM nocodazole and prepared for immunofluorescence. Ska3 at kinetochores was quantified. (D) Plk1 or BubR1 depletion reduced Ska3 at kinetochores. (E) HeLa H2B-GFP cells were transfected with PP1γ or PP2A Aα siRNA individually or in combination at 50 nM final concentration. Approximately 30 h post transfection, mitotic progression was followed by video microscopy. Bar graph depicts mean chromosome alignment times. PP1 depletion causes a slight delay in alignment. PP2A Aα depletion shows a stronger delay while combined depletion of PP1γ and PP2A Aα shows the most robust delay. (F) Bar graph depiction of time taken to initiate anaphase in individual cells. PP1γ or PP2A Aα depletions either individually or in combination delay mitotic progression. The combined depletions are more penetrant suggesting a compensatory or redundant role for the phosphatases when depleted individually. (G) Bar graph showing percentage of siRNA-treated cells arrested in metaphase and undergoing cohesion fatigue after depleting PP1γ, PP2A Aα or both. Graphs B, D-F show mean±s.e.m., unpaired Student's *t*-test, two-tailed distribution.

We and others have shown that Ska binds to kinetochores at prometaphase and maximally accumulates there at metaphase (Chan et al., 2012; Redli et al., 2016; Sivakumar et al., 2014). Inhibition of Aurora B kinase increased Ska accumulation on kinetochores lacking microtubule attachment (Chan et al., 2012). Correspondingly, expression of phosphomimetic mutants of Ska inhibited recruitment (Chan et al., 2012). These findings and recent data from Redli et al. (2016) indicate phosphatases likely regulate Ska binding to kinetochores. PP1 and PP2A are the major phosphatases implicated in mitotic transitions. PP1, principally the PP1γ isoform, localizes to kinetochores and is implicated in spindle checkpoint inactivation (Liu et al., 2010; Trinkle-Mulcahy et al., 2003). PP2A also accumulates at kinetochores and plays a role in promoting kinetochore-microtubule attachment in prometaphase (Foley et al., 2011). To test the role of the phosphatases in Ska recruitment, we depleted PP1γ or PP2A Aα subunit. We analyzed recruitment of Ska to kinetochores using immunofluorescence with antibody to Ska3. Ska begins to concentrate at kinetochores before microtubule attachment but reaches maximum levels on bioriented metaphase chromosomes. In cells progressing through mitosis with intact spindles, we found that depletion of PP1γ or PP2A Aα reduced Ska3 kinetochore levels (Fig. S1A-C). However, depletion of phosphatases has direct effects on spindle microtubule stability (Foley et al., 2011; Liu et al., 2010). To eliminate the complication of varying spindle microtubule stability after depletion of phosphatases in experiments designed to quantify Ska accumulation on kinetochores, we measured Ska levels on kinetochores of nocodazole-treated cells and found that depletion of PP1 or PP2A phosphatase significantly decreased Ska3 accumulation (Fig. 1A,B). Previous work has shown that Plk1 and BubR1 promote PP2A recruitment to kinetochores (Foley et al., 2011; Suijkerbuijk et al., 2012). Depletion of Plk1 or BubR1 with siRNA caused the expected reduction of PP2A at kinetochores in cells with intact spindle microtubules (Fig. S1D-F) and also resulted in lower levels of kinetochore-associated Ska3 in nocodazole-treated mitotic cells (Fig. 1C,D).

Previous studies have shown that depletion of various isoforms and subunits of PP1 and PP2A phosphatases can induce defects in mitotic chromosome movements, and delays in silencing the spindle checkpoint and promoting the metaphase-anaphase transition (Foley et al., 2011; Grallert et al., 2015; Lee et al., 2017; Liu et al., 2010; Tang et al., 2006). These phenotypes are reminiscent of those seen after Ska depletion but generally milder, likely because PP1 and PP2A exist as multiple isoforms such that depletions generally affect only a subset of phosphatase activities. Nevertheless, since we found that PP1 and PP2A appear to promote Ska accumulation at kinetochores, we tested if depletion of the phosphatase subunits in our study generated similar phenotypes. We found that depletion of PP1γ or PP2A Aα caused small delays in chromosome alignment and in the metaphase-anaphase transition (Fig. 1E,F). Combined depletions of both PP1γ and PP2A Aα showed the strongest effects. While we could reproducibly generate mitotic defects upon phosphatase depletions, we were unable to rescue these by expressing RNAi-resistant plasmids, despite several attempts. This may be due to failure of tagged exogenous constructs to effectively complement endogenous proteins, inability to precisely match the level of depletion of specific isoforms, or other technical variables we were unable to control. However, given our inability to rescue the phenotypes, we cannot formally exclude the possibility that our observed phenotypes were influenced by potential off-target effects of the siRNAs.

**Fig. 2.**
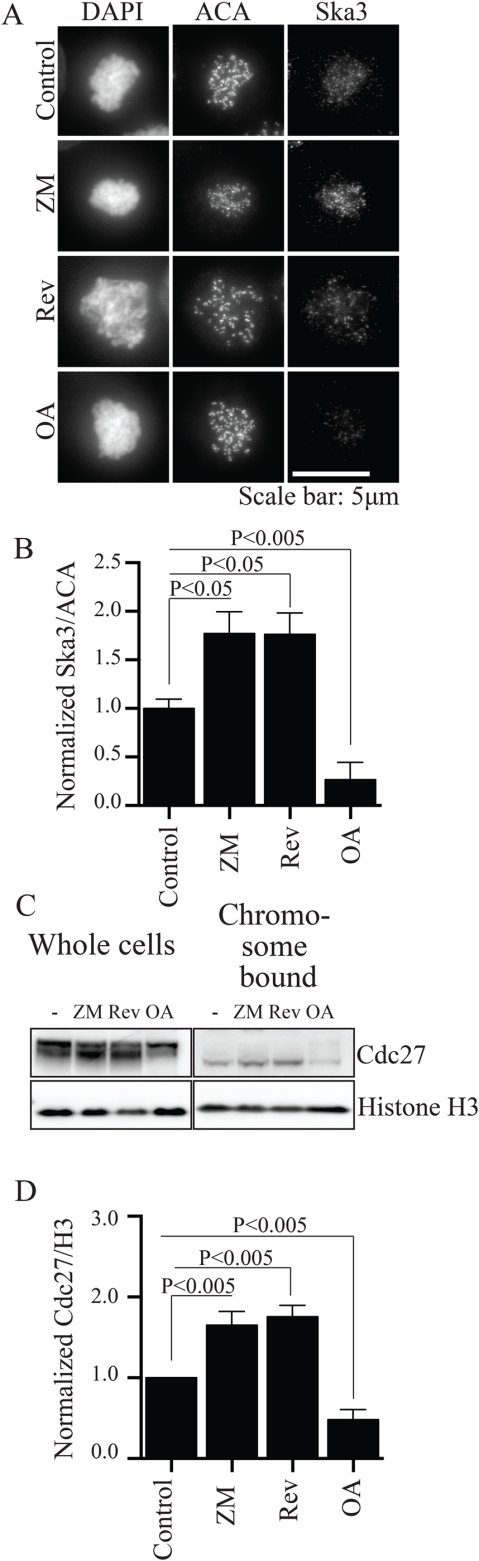
**Kinase and phosphatase inhibitors regulate Ska recruitment to kinetochores and APC/C recruitment to mitotic chromosomes.** (A) HeLa cells grown on glass coverslips were treated with 3.3 µM nocodazole and 25 μM MG132 for 2 h. Coverslips were individually treated with DMSO (control), 1μM Reversine (Rev, Mps1 inhibitor), 25 µM ZM447439 (ZM, Aurora B inhibitor) or 0.5 μM Okadaic acid (OA, phosphatase inhibitor) for an additional 1 h. Cells were pre-extracted, fixed and labeled with anti-Ska3 and ACA antibodies, then processed for immunofluorescence. (B) Quantification shows that Aurora B or Mps1 inhibition increased Ska3 at kinetochores while phosphatase inhibition decreased Ska3 at kinetochores. (C) HeLa cells, treated as in A, were collected. A portion was lysed directly into sample buffer (whole cells) and the rest used to prepare isolated mitotic chromosomes (chromosomes). Western blots were labeled with antibodies to histone H3 and Cdc27, a component of the APC/C. In whole cell lysate, most Cdc27 is hyperphosphorylated and shows slower mobility. That bound to mitotic chromosomes shows lower phosphorylation and higher electrophoretic mobility. (D) Quantification of western blots shows that like Ska, Cdc27 levels increase in chromosomes treated with Aurora B or Mps1 inhibitors and decrease in OA-treated cells. Graphs B and D show mean±s.e.m., unpaired Student's *t*-test, two-tailed distribution.

Therefore, as an additional approach to test the roles of phosphatases and kinases in Ska kinetochore recruitment, we used small molecule kinase and phosphatase inhibitors to examine effects on Ska recruitment to kinetochores. HeLa cells were treated with these drugs in nocodazole to depolymerize spindle microtubules and in proteasome inhibitor, MG132, to block mitotic exit. As reported previously, inhibition of Aurora kinase increased recruitment of Ska to kinetochores (Chan et al., 2012). However, in contrast to previous findings we found that treatment of cells with an inhibitor of Mps1 also increased Ska recruitment (Fig. 2A,B) (Chan et al., 2012). This finding is consistent with roles for Mps1 in recruitment of Aurora B and BubR1 to kinetochores (Krenn et al., 2014; Overlack et al., 2015; van der Waal et al., 2012; Zhang et al., 2014). As also reported in a recent study (Redli et al., 2016), we found that treatment of cells with the phosphatase inhibitor, okadaic acid, decreased Ska accumulation at kinetochores (Fig. 2A,B), consistent with results from our RNAi depletion of phosphatases described above.

Previously we found that Ska complex promotes APC/C accumulation on chromosomes purified from mitotic cell extracts (Sivakumar et al., 2014). We tested if kinase or phosphatase inhibitors that affected kinetochore concentration of Ska had similar effects on chromosome-bound APC/C prepared from cell extracts arrested with nocodazole. Indeed, we found that inhibitors of Aurora B or Mps1 increased APC/C on chromosomes while the phosphatase inhibitor, okadaic acid, decreased APC/C on chromosomes (Fig. 2C,D).

### Microtubule attachment and PP1 promote Ska3 kinetochore recruitment

**Fig. 3.**
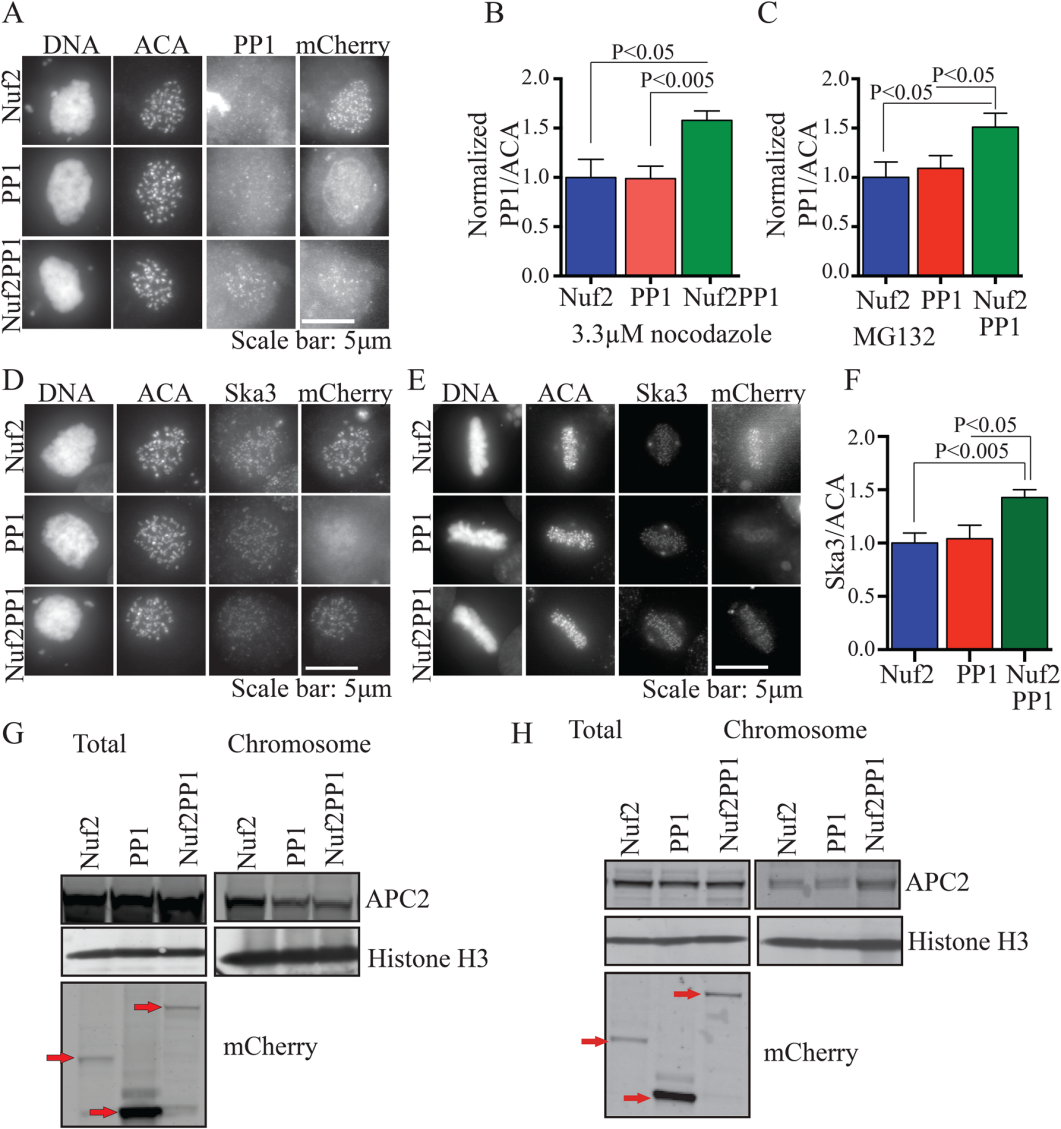
**Expression of a Nuf2PP1 fusion in the presence of intact spindle microtubules promotes Ska recruitment to kinetochores and APC/C recruitment to chromosomes.** (A) HeLa cells grown on coverslips were transfected with Nuf2-mCherry, PP1-mCherry or Nuf2PP1-mCherry. 36 h after transfection 3.3 μM nocodazole was added to cells for 3 h and cells were prepared for immunofluorescence. (B) PP1 localization to kinetochores was quantified. Nuf2PP1 expression increased PP1 at kinetochores by 50% compared to controls. (C) HeLa cells transfected with Nuf2-mCherry, PP1-mCherry or Nuf2PP1-mCherry were released from 330 nM nocodazole into MG132 for 2 h. Cells were prepared for immunofluorescence and PP1 at kinetochores was measured. (D) HeLa cells were treated as in A and B, then prepared for immunofluorescence. In cells arrested in nocodazole, additional PP1 at kinetochores did not measurably increase recruitment of Ska3. (E) HeLa cells were treated as in C and Ska3 at kinetochores was measured. In cells released from nocodazole and allowed to progress to metaphase in MG132, additional PP1 at kinetochores increased recruitment of Ska3. (F) Quantification of Ska3 levels in metaphase arrested cells expressing Nuf2-mCherry, PP1-mCherry and Nuf2PP1-mCherry. (G) Cells were treated as in A and C and then used to prepare whole cell lysate (Total) and mitotic chromosomes (Chromosome). These samples were then blotted for APC2, Histone H3 and for mCherry to reveal expression levels of the transgenes. In nocodazole, targeting PP1 to kinetochores did not significantly increase the amount of chromosome-associated APC/C. Chromosome bound APC/C is similar in cells expressing Nuf2-mCherry, PP1-mCherry, or Nuf2PP1-mCherry. (H) Cells were treated as in C and then used to prepare whole cell lysate (Total) and mitotic chromosomes (Chromosome). In metaphase cells, targeting PP1 to kinetochores increases the level of chromosome-associated APC/C. Quantification of this result is depicted in Fig. S2C. Graphs B, C and F show mean±s.e.m., unpaired Student's *t*-test, two-tailed distribution.

We previously demonstrated that an important function of the C-terminus of Ska1 is to recruit PP1 to kinetochores (Sivakumar et al., 2016). In those experiments, Ska2, Ska3 and the N terminus of Ska1 and their potential separate functions were retained. To study the effects of forced targeting of PP1 in the complete absence of Ska components, we constructed a fusion of the outer kinetochore protein, Nuf2, to PP1. Nuf2 is a component of the Ndc80 complex, which is implicated in recruiting Ska to kinetochores (Chan et al., 2012; Zhang et al., 2012; Zhang et al., 2017). We reasoned that the Nuf2PP1 fusion would target PP1 to a region of the kinetochore near to the normal Ska-recruited PP1. Expression of the Nuf2PP1 fusion induced a significant increase in kinetochore-associated PP1, both in cells arrested in a prometaphase-like state in nocodazole and at metaphase in MG132 (Fig. 3A,B,C), even though the Nuf2PPI fusion was expressed at lower levels than unfused Nuf2 or PP1 in controls (Fig. S2A). We then analyzed Ska recruitment. In nocodazole-treated cells, we detected little effect of the Nuf2PP1 fusion on Ska accumulation at kinetochores (Fig. 3D; Fig. S2B). This may be due to the greater difficulty of detecting changes in the low levels of Ska found at kinetochores of nocodazole-treated cells or from the inability of the Nuf2PP1 fusion to perfectly mimic the normal recruitment of PP1. However, in MG132-treated cells at metaphase, where Ska levels are higher, we measured a 50% increase in Ska accumulation at kinetochores in cells expressing the Nuf2PP1 fusion (Fig. 3E,F). We then examined the effect of Nuf2PP1 expression on binding of the APC/C to mitotic chromosomes (Sivakumar et al., 2014). Consistent with the findings above on the effects of Nuf2PP1 on Ska recruitment, we found that Nuf2PP1 increased APC/C on chromosomes isolated from cells arrested at metaphase in MG132 but not from chromosomes obtained from cells arrested in nocodazole (Fig. 3G,H; Fig. S2C).

### Expression of Nuf2PP1 fusion partially rescues mitotic defects in Ska3-depleted cells

**Fig. 4.**
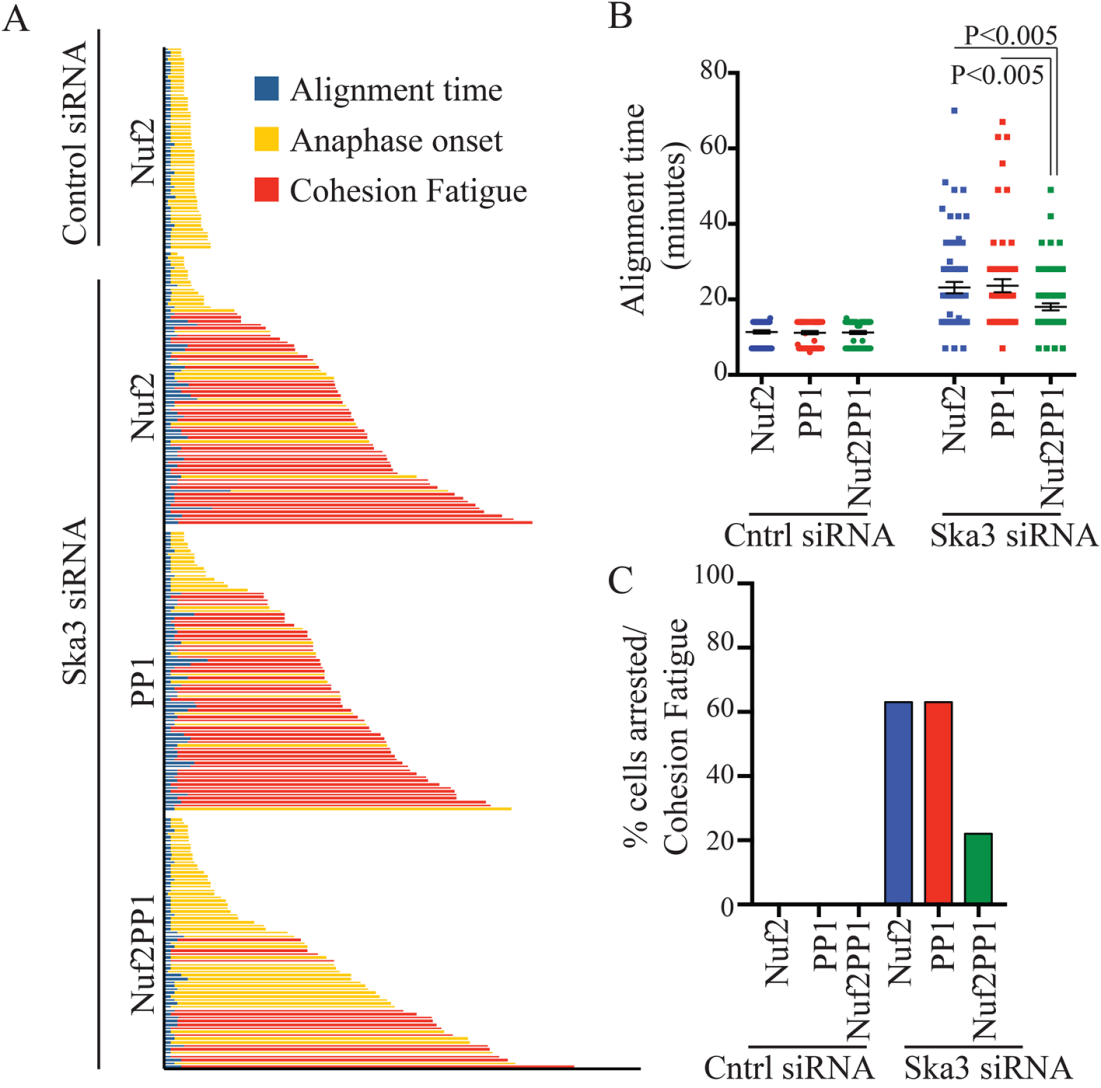
**Expression of Nuf2PP1 fusion partially rescues mitotic defects caused by Ska depletion.** (A) Tracking of fates for individual HeLa cells stably expressing histone H2B-GFP and transfected with Nuf2-mCherry, PP1-mCherry or Nuf2PP1-mCherry. 12 h after transfection, cells were treated with control or Ska3 siRNA. Time-lapse imaging was initiated 24 h after siRNA transfection. The mCherry constructs did not affect cells transfected with control siRNA so only the Nuf2-mCherry control is shown here. The other controls are summarized in Fig. S2D. The time intervals to align chromosomes, initiate anaphase or undergo metaphase arrest/cohesion fatigue were measured. Expression of Nuf2-PP1 partially rescued alignment times, metaphase delays, and cohesion fatigue induced by Ska3 depletion. (B) Time intervals for chromosome alignment in control siRNA or Ska3 siRNA for Nuf2-mCherry, PP1-mCherry or Nuf2PP1-mCherry expressing cells. Expression of Nuf2PP1-mCherry partially reduced alignment in Ska3-depleted cells. Horizontal lines indicate mean and whiskers indicate standard error. *P* values were determined using the Mann–Whitney test. (C) The percentages of cells that arrested at metaphase for the duration of imaging and/or underwent cohesion fatigue in control or Ska3-depleted cells expressing Nuf2-mCherry, PP1-mCherry or Nuf2PP1-mCherry. In Ska3-depleted cells, expression of Nuf2PP1 increased the proportion of cells that underwent anaphase onset compared to expression of Nuf2 or PP1.

Depletion of any Ska component results in delayed alignment followed by a robust mitotic delay or arrest at metaphase (Daum et al., 2009; Redli et al., 2016; Schmidt et al., 2012; Sivakumar et al., 2014). The metaphase delay often results in cohesion fatigue, asynchronous separation of chromatids without mitotic exit (Daum et al., 2011; Sivakumar et al., 2014; Stevens et al., 2011). We tested if Nuf2PP1 expression rescued mitotic defects in Ska-depleted cells. In control cells, expression of Nuf2, PP1 or Nuf2PP1 did not alter mitosis (Fig. S2D). In Ska3-depleted cells, expression of the Nuf2PP1, but not Nuf2 or PP1 alone, improved alignment (Fig. 4A,B). In addition, while 63% of Ska-depleted cells expressing Nuf2 or PP1 arrested at metaphase and underwent cohesion fatigue, only 22% of cells expressing the Nuf2PP1 fusion did so (Fig. 4C). However, the rescue was incomplete. Most Ska3-depleted cells expressing the Nuf2PP1 fusion still exhibited a significant delay before entering anaphase (Fig. 4A). The failure of the Nuf2PP1 fusion to fully rescue Ska depletion is consistent with the idea that the entire Ska complex has important functions in addition to PP1 recruitment. There are also potential technical reasons why the rescues were incomplete. Both PP1 and Ska have been shown to exhibit rapid turnover at kinetochores (Raaijmakers et al., 2009; Trinkle-Mulcahy et al., 2001). In contrast, the Ndc80 complex, of which Nuf2 is a component, is stable (Hori et al., 2003). Thus, Nuf2PP1 likely fails to exhibit the normal dynamics of PP1 and this limitation and other technical limitations of the Nuf2PP1 fusion may prevent complete rescue of Ska depletion phenotypes.

### Conclusions

Here we show that PP1 and PP2A are required for full accumulation of Ska on kinetochores. There is significant interplay between PP1 and PP2A phosphatases at kinetochores to allow precise spindle checkpoint signaling and to modulate proper microtubule attachments (Grallert et al., 2015; Nijenhuis et al., 2014). Upon proper microtubule attachment, kinetochore PP1 displaces PP2A to allow spindle checkpoint silencing (Nijenhuis et al., 2014). Our data indicate that both PP1 and PP2A are required for recruitment of Ska to kinetochores. Further work will be required to determine if either phosphatase directly mediates the accumulation of Ska at kinetochores.

Though cells depleted of PP1γ and PP2A Aα show alignment defects and metaphase delays, they rarely exhibit the strong phenotypes characteristic of Ska depletions (Fig. 1G). There are several potential explanations for this observation. PP1 and PP2A proteins may be long-lived or be sufficient at low protein concentration. For PP1 we targeted only PP1γ. Other isoforms may compensate, particularly PP1α, which also accumulates at kinetochores. For PP2A we targeted the Aα scaffolding subunit but cells also express the Aβ scaffolding subunit. Other protein phosphatases beyond PP1 and PP2A may also participate in mitotic regulation (Della Monica et al., 2015; Huang et al., 2016; Zeng et al., 2010).

If PP1 recruitment were the exclusive function of Ska, expression of Nuf2PP1 in Ska-depleted cells might be expected to completely alleviate phenotypes caused by reduction of Ska levels. We found only partial rescue. Most likely, PP1 recruitment, a function of the C terminus of Ska1, reflects only one function of the full Ska complex. Additional roles such as microtubule tracking may also promote alignment and timely metaphase-anaphase transition (Abad et al., 2014, 2016; Chan et al., 2012; Gaitanos et al., 2009; Raaijmakers et al., 2009; Redli et al., 2016; Schmidt et al., 2012; Welburn et al., 2009).

PP1 has been shown to be integral in opposing spindle checkpoint signaling and is required to promote normal anaphase onset (Liu et al., 2010; Sivakumar et al., 2016). PP1 levels at kinetochores increase from prometaphase to metaphase. We speculate that low levels of PP1 and Ska at kinetochores and APC/C on chromosomes in prometaphase prevent premature anaphase onset and mitotic exit. Then, at metaphase, Ska binding to microtubules increases its kinetochore concentration creating a positive feedback loop whereby PP1 accumulation at kinetochores further increases Ska leading to accumulation of kinetochore PP1 and chromosome APC/C. This feedback strengthens microtubule attachment, opposes spindle checkpoint signaling and promotes the rapid and irreversible transition to anaphase and mitotic exit.

## MATERIALS AND METHODS

### Cell culture

HeLa cells stably transfected with GFP fused to Histone 2B (HeLa H2B-GFP) were used in this study. HeLa cell lines were grown in culture flasks or chambered coverslips in DMEM-based media with 10% FBS supplemented with penicillin and streptomycin in 5% CO_2_ at 37°C. Cell lines were routinely tested for mycoplasma contamination and only used if not contaminated.

Transient transfection of siRNA was done using Lipofectamine RNAi reagent (Invitrogen) according to manufacturer's instructions. siRNA against PP2A Aα (CACAGAGAAAUAAAGGUCU and ACAACGUCAAGAGUGAGAU), PP1γ (CGAGUGACCGAUUAUGCUU and GUCUGAGGAGUAAGUGUAC) were obtained from Bioneer Inc. (Almeda, CA, USA) and these were used at 50-100 nM final concentration. siRNA against Ska3 was obtained from Dharmacon (Lafayette, CO, USA) and used at 50 nM final concentration (Daum et al., 2009).

### Plasmids

Nuf2, PP1 or Nuf2PP1-mCherry plasmids were constructed by inserting respective cDNA in mCherry-N1 vector. mCherry tag was inserted upstream or downstream of transgene and similar results were obtained with proteins tagged in N or C termini.

### Live cell imaging

HeLa H2B-GFP cells were grown in Nunc chambered coverslips (Thermo Fisher Scientific, Waltham, MA, USA). To maintain appropriate pH levels and avoid evaporation during imaging, culture media was exchanged to Leibovitz's L-15 medium supplemented with 10% FBS, penicillin, streptomycin and overlaid with mineral oil. Time-lapse fluorescence images were collected using 20× or 40× objectives and a Zeiss Axiovert 200 M inverted microscope equipped with an objective heater, air curtain, a Hamamatsu ORCA-ER camera, and Metamorph software (Molecular Devices, Sunnyvale, CA, USA). Images were captured every 4–15 min for 12–24 h. Time-lapse videos displaying the elapsed time between consecutive frames were assembled using Metamorph software. The mitotic interval was calculated from nuclear envelope breakdown (NEB) until anaphase onset/mitotic exit and depicted as bar graphs or scatter plots with mean and s.e.m. In scatter plots, each dot represents one cell; long horizontal line depicts mean and whiskers denote s.e.m. The unpaired Student *t*-test or Mann–Whitney test were performed in Graphpad Prism (Graphpad Software, La Jolla, CA, USA) to assess statistical significance.

### Immunofluorescence and quantification

HeLa cells were grown on glass coverslips and treated as detailed in the figure legends. Cells were pre-extracted in PHEM/1% triton solution for 5 min before fixing with 1.5% paraformaldehyde/PHEM solution for 15 min. Coverslips were washed in MBST, blocked in 20% boiled goat or donkey serum, and incubated overnight with primary antibodies. Samples were then incubated with secondary antibodies for 1-2 h, stained with DNA dye, DAPI, and mounted using Vectashield (Vector Laboratories, Burlingame, CA, USA). The following primary antibodies were used: rabbit anti-Ska3 (Daum et al., 2009), ACA/CREST (Antibody Inc., Davis, CA, USA), mouse anti-Bub1 (antibody 4B12 from Dr Steven Taylor, University of Manchester), rabbit anti-Plk1 (Upstate, Lake Placid, NY, USA), rabbit anti-BubR1 (gift from Dr Todd Stukenberg), goat anti-PP1γ (Santa Cruz Biotechnology, Dallas, TX, USA), goat anti-PP2A Aα/β (Santa Cruz Biotechnology). Secondary antibodies used were goat anti–rabbit, goat anti–mouse, donkey anti-goat antibodies conjugated to Cy3 or FITC or goat anti-human antibody conjugated to Cy3 or FITC (Jackson ImmunoResearch, West Grove, PA, USA). The images were acquired using Zeiss Axioplan II microscope equipped with a 100× objective (N.A. 1.4), a Hamamatsu Orca 2 camera (Hamamatsu Photonics, Bridgewater, NJ, USA) and processed using MetaMorph software and Coreldraw (Corel Corp., Ottawa, ON, Canada). Quantification of the immunofluorescence images was done as described previously (Daum et al., 2009; Sivakumar et al., 2014). 5-10 cells in each condition were quantified. The graphs depict average fluorescence value with s.e.m. in each condition. Every experiment was repeated at least three times and representative images are shown.

### Western blotting

Whole cell HeLa cell extracts were prepared by lysis in APCB buffer (20 mM Tris-Cl pH 7.7, 100 mM KCl, 50 mM Sucrose, 1 mM MgCl2, 0.1 mM CaCl2, 0.5% Triton X-100) containing protease inhibitor cocktail (Sigma Aldrich, St Louis, MO, USA) and microcystin (400 nM). For electrophoresis, sample loading buffer (Invitrogen) and dithiothreitol (DTT) to a final concentration of 50 mM were added. Proteins were separated with a NuPAGE gel electophoresis system (Invitrogen, Thermo Fisher Scientific), transferred to 0.45 µm PVDF membrane (Immobilon PVDF, Millipore, Burlington, CA, USA) via a Genie transfer apparatus (Idea Scientific, Minneapolis, MN, USA). Membranes were blocked in 5% Non-Fat Dry Milk (NFDM) and 0.05% Tween 20 in Tris-buffered saline (TBS). Primary antibodies included rabbit anti-Ska3 antibody (Daum et al., 2009), mouse anti-β-Actin (Abcam, Cambridge, UK), anti-Plk1 (Upstate), anti-BubR1 (gift from Dr Todd Stukenberg). Membranes were washed in TBS/0.05% Tween 20 (TBST), and then incubated with secondary antibodies in 5% NFDM/TBST. Secondary antibodies include HRP goat anti-mouse or anti-rabbit antibodies (Jackson Immunoresearch). After washes, membranes were developed using West Pico Chemiluminescent reagent (Pierce, Rockford, IL, USA) and imaged using a Kodak 4000 M imaging station. Quantification of western blots was done as described previously (Sivakumar et al., 2014, 2016). Every experiment was repeated at least three times.

### Chromosome preparation

HeLa cells were grown in 150 mm plates and treated as indicated in figure legends. Mitotic cells were lysed with ELB buffer on ice (1× PHEM, 0.5% Triton X-100, 1 mM DTT, 10% glycerol, protease inhibitors and 400 nM microcystin) and centrifuged to separate cytoplasmic fractions from chromosome fractions. Chromosome fractions were further washed with ELB at least three times to remove cytoplasmic contamination and resuspended in 1/4th volume ELB of cytoplasmic fractions. The protein concentration of cytoplasmic fractions was determined using the BCA protein assay kit (Pierce). The chromosomes were DNAse treated and resuspended in sample loading buffer (Invitrogen) and 50 mM DTT. Samples were immunoblotted with antibodies to the APC/C components Cdc27 and APC2 and antibody to Histone H3.

## Supplementary Material

Supplementary information

First Person interview
