# Supplementary material for: Phosphatase-regulated recruitment of the spindle- and kinetochore-associated (Ska) complex to kinetochores
Source: Biol Open. 2017 Oct 5;6(11):1672–9. doi: 10.1242/bio.026930 (PMC5703607; doi:10.1242/bio.026930)
Supplement: Supplementary information [file biolopen-6-026930-s1.pdf]

## SUPPLEMENTAL FIGURES

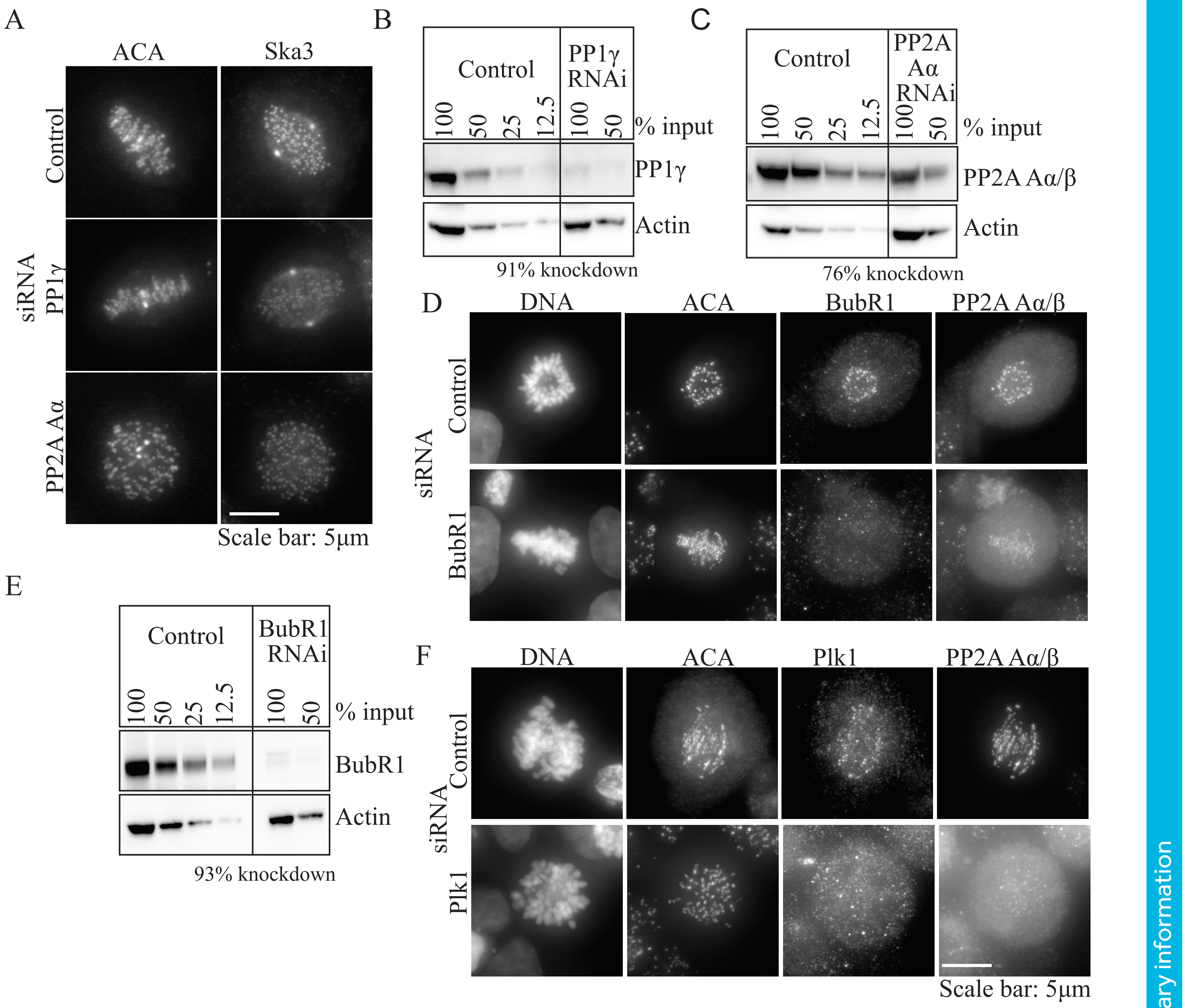

**Figure S1: Characterization of siRNA depletions.** A) Normally cycling HeLa cells were treated with control, PP1γ or PP2A Aα siRNA for 48h. Cells were fixed and prepared for immunofluorescence to determine effects on Ska3 recruitment to kinetochores. Depletion of PP1γ or PP2A Aα caused a noticeable reduction in the level of Ska3 at kinetochores. B) HeLa cells transfected with PP1γ siRNA were arrested in mitosis with 3.3μM nocodazole and collected. Western blotting of whole cell lysates indicated that PP1γ was depleted by 91%. C) HeLa cells transfected with PP2A Aα siRNA were arrested in mitosis with 3.3μM nocodazole and collected. Western blotting of whole cell lysates indicated that labeling with antibody that detects PP2 Aα and PP2A Aβ was reduced by 76%. It is uncertain how much of the residual label reflects expression of PP2A Aβ versus incomplete depletion of PP2A Aα. D) HeLa cells transfected with control or BubR1 siRNA. Immunofluorescence was done and cells were labelled with antibody to BubR1 and PP2A Aα/β. BubR1 depletion reduced BubR1 accumulation at kinetochores by approximately 75% and PP2A Aα/β by approximately 40%. E) HeLa cells transfected with BubR1 siRNA were arrested in mitosis with 3.3μM nocodazole and collected. Western blotting of whole cell lysates indicated that BubR1 was depleted by 93%. F) HeLa cells were transfected with control or Plk1 siRNA. Cells were immunolabeled with Plk1 and PP2A Aα/β. PP2A Aα/β was reduced by approximately 50% in Plk1 depleted cells.

Biology Open • Supplementary information

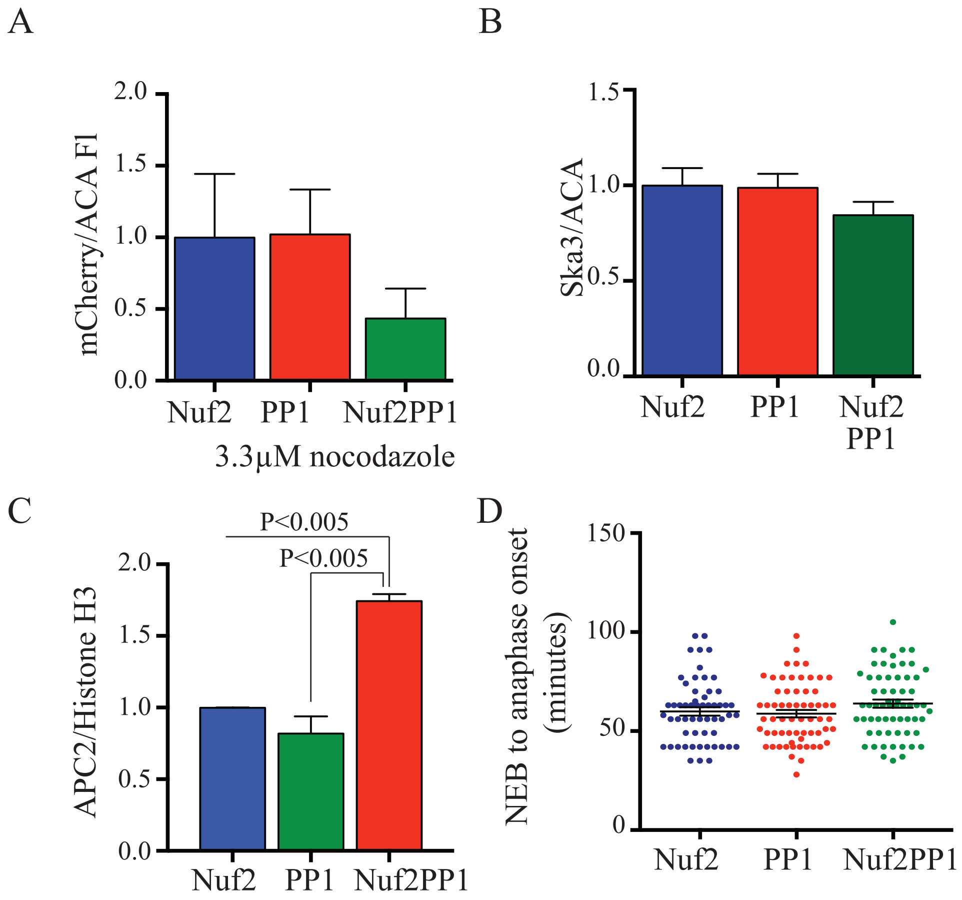


**Figure S2: Expression of Nuf2PP1 does not increase kinetochore-associated Ska in nocodazole-arrested cells but does increase APC/C on chromosomes in MG132-arreseted cells.** A) HeLa cells were transfected with Nuf2-mCherry, PP1-mCherry or Nuf2PP1-mCherry. mCherry levels were quantified. Nuf2PP1 expression was lower than Nuf2 or PP1 expression. B) In cells arrested with nocodazole, Ska3 at kinetochores was not increased in cells expressing Nuf2-mCherry, PP1-mCherry, or Nuf2PP1-mCherry. C) Cells expressing Nuf2-mCherry, PP1-mCherry, or Nuf2PP1-mCherry were arrested in metaphase with MG132. These were used to prepare whole cell lysates and chromosome fractions which were blotted for APC2 and Histone H3. Representative images of the results from three repeats are shown in Figure 3H. Metaphase cells expressing Nuf2-PP1 show higher levels of the APC/C component, APC2, bound to mitotic chromosomes. D) HeLa cells transfected with Nuf2-mCherry, PP1-mCherry or Nuf2PP1-mCherry were imaged by time-lapse microscopy. Mitotic progression is similar in cells expressing Nuf2-mCherry, PP1-mCherry, or Nuf2PP1-mCherry.

Biology Open • Supplementary information
