# Supplementary material for: Phosphatase-regulated recruitment of the spindle- and kinetochore-associated (Ska) complex to kinetochores
Source: Biol Open. 2017 Oct 5;6(11):1672–9. doi: 10.1242/bio.026930 (PMC5703607; doi:10.1242/bio.026930)
Supplement: First Person interview [file biolopen-6-026930-s2.pdf]

FIRST PERSON

First person – Sushama Sivakumar

First Person is a series of interviews with the first authors of a selection of papers published in Biology Open, helping early-career researchers promote themselves alongside their papers. Sushama Sivakumar is first author on 'Phosphatase-regulated recruitment of the spindle- and kinetochore-associated (Ska) complex to kinetochores', published in BiO. Sushama conducted the research in this article while a PhD student in Gary J. Gorbsky's lab at the Oklahoma Medical Research Foundation, USA, but is now a postdoctoral researcher at the University of Texas Southwestern Medical Center, investigating regulatory mechanisms that control mitotic progression and allow for the accurate segregation of chromatids during the cell cycle.

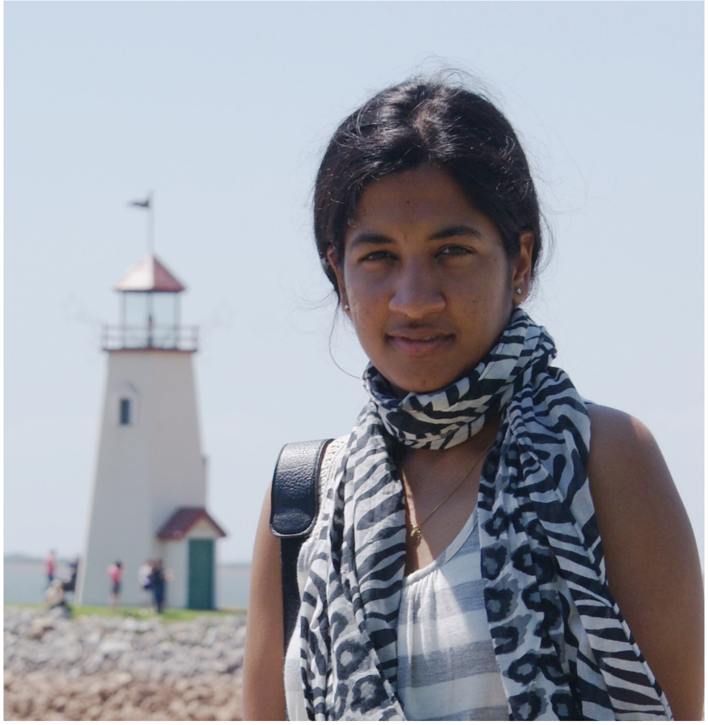

Sushama Sivakumar

What is your scientific background and the general focus of your lab?

We are interested in studying the regulatory mechanisms that ensure proper chromosome segregation during mammalian cell mitosis. During my PhD in Gary Gorbsky's lab, we were involved in studying the functions of a novel protein complex called Ska (spindle- and kinetochore-associated) in mitotic progression. Depletion of Ska proteins causes chromosome alignment defects often followed by a penetrant metaphase arrest that ultimately results in cohesion fatigue. My PhD project elucidated how Ska proteins function in metaphase–anaphase transition and contribute to both spindle checkpoint signalling and APC/C activation.

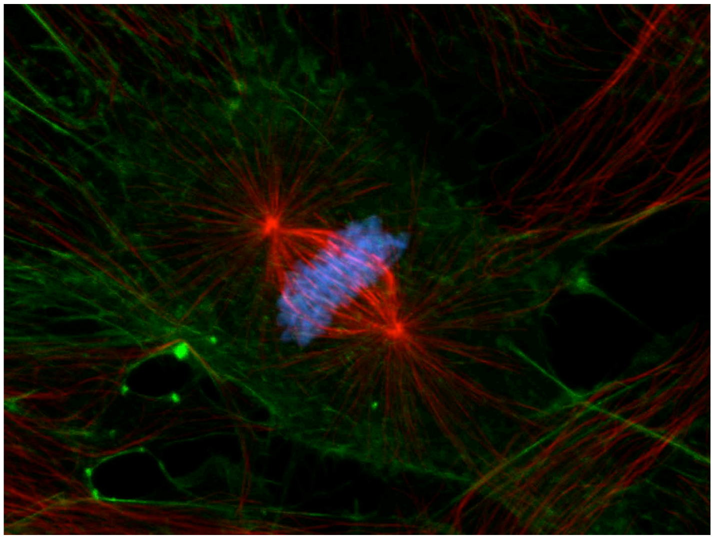

A metaphase cell with chromosomes (blue) perfectly aligned on the metaphase plate and attached to microtubules (red).

Sushama Sivakumar's contact details: Department of Pharmacology, University of Texas Southwestern Medical Center, Dallas, TX 75390, USA.

E-mail: Sushama.Sivakumar@UTSouthwestern.edu

How would you explain the main findings of your paper to non-scientific family and friends?

Our paper discusses a novel regulatory mechanism that cells use to couple microtubule attachment to spindle checkpoint signalling during cell division. The spindle checkpoint is an evolutionary conserved mechanism that prevents chromatid separation until all chromosomes (condensed DNA) are attached to microtubules (thread-like fibres that bind and move chromosomes). The kinetochore is a multi-subunit protein complex that forms on chromosomes and functions in microtubule binding and chromosome movement. A novel protein complex called Ska monitors microtubule attachment to kinetochores and upon proper attachment recruits other proteins that signal the spindle checkpoint to promote anaphase onset. Our paper details the proteins required to localise Ska at kinetochores. At metaphase, proper microtubule attachments cause accumulation of Ska protein at kinetochores that leads to a feedforward loop that further stabilises microtubule attachments and inactivation of the spindle checkpoint. This ensures that spindle checkpoint silencing occurs only after microtubule attachments are properly made. This safety check is absolutely required to guarantee that the daughter cells have the correct DNA content once the cell has divided.

What are the potential implications of these results for your field of research?

Proper chromosome attachment and irreversible onset of anaphase are required to maintain genome integrity. Problems in the process cause aneuploidy or abnormal chromosome number that is detrimental to cells. Our paper unravels an important regulatory mechanism that

ensures that spindle checkpoint inactivation occurs only after stable microtubule attachments are made. If spindle checkpoint signalling is erroneous and mitotic exit occurs before proper microtubule attachments are made, daughter cells end up with incorrect amount of DNA. This causes protein imbalances in the daughter cells leading to problems in the cell cycle. Our study helps us understand how microtubule attachment may be coupled to spindle checkpoint signalling to prevent abnormal DNA content in daughter cells. Elucidating this mechanism will help develop pharmaceutical drugs that can target dividing cancerous cells and treat tumours.

What has surprised you the most while conducting your research?

The most surprising part of researching the cell cycle is how cells go through the complex process in a well-coordinated and regulated manner. There are many safety checks that ensure that the next stage does not occur until proper completion of a previous stage. This makes sense when we think about it and sounds easy, but for a cell to coordinate its many molecules to ensure that the right interaction occurs at the right place at the right time every cell cycle is pretty amazing! If there are some defects or errors in the process, cells have different ways to fix it. If those fail then ultimately a cell can trigger its own death. Such regulation and safety mechanisms in a biological system is what makes studying the cell cycle very fascinating.

What, in your opinion, are some of the greatest achievements in your field and how has this influenced your research?

Signalling pathways in science have always fascinated me. The discovery of the regulatory pathways that precisely control the irreversible transitions in the cell cycle are very interesting. Both mitotic entry and mitotic exit are well coordinated to ensure accuracy and unidirectionality. Studies that improved our understanding of kinetochore components, spindle checkpoint signalling pathways and microtubule motors were all key discoveries that helped me comprehend my research better. We were studying a unique protein complex that localised to distinct sub-cellular compartments during mitosis and wanted to investigate its myriad functions. Regular reading and experimentation in new directions really helped us unravel some of the mitotic roles of this protein complex. My PhD mentor also encouraged us to attend relevant national and international meetings. At these meetings, I was fortunate to interact with peers and senior scientists who gave me valuable advice and feedback that was integral to the advancement of our studies.

“Regular reading and experimentation in new directions really helped us unravel some of the mitotic roles of this protein complex.”

What changes do you think could improve the professional lives of early-career scientists?

As a postdoctoral researcher I am given freedom, financial support and guidance to grow as a scientist. I am not a principal investigator yet but from talking to peers and other scientists who have recently got the opportunity to start their own laboratories, I think early-career scientists can profit from getting more advice and support from peers. Sharing experiences and advice on how to tackle new situations or new personnel can be valuable at this stage in their careers.

What’s next for you?

I have enjoyed doing research over the last 10 years. I still love working at my bench, doing experiments and writing about our studies. I hope I can continue to do this for a very long time.

Reference

Sivakumar, S. and Gorbsky, G. J. (2017). Phosphatase-regulated recruitment of the spindle- and kinetochore-associated (Ska) complex to kinetochores. *Biol. Open* **6**, doi:10.1242/bio.026930.
